# Maternal Separation and Negative Renal Programming, Evidence of Morphofunctional Alterations in Rodent Models: Systematic Review and Meta-Analysis

**DOI:** 10.3390/ijms262110509

**Published:** 2025-10-29

**Authors:** Jhonatan Duque-Colorado, Josue Rivadeneira, Bélgica Vásquez

**Affiliations:** 1 Doctoral Program in Morphological Sciences, Faculty of Medicine, Universidad de La Frontera, Temuco 4811230, Chile; jhonatanandresduquecolorado@gmail.com; 2 Center of Excellence in Morphological and Surgical Studies, Universidad de La Frontera, Temuco 4811230, Chile; 3 Doctoral Program in Medical Sciences, Universidad de La Frontera, Temuco 4811230, Chile; j.rivadeneira01@ufromail.cl; 4 Zero Biomedical Research, Quito 170403, Ecuador; 5 Department of Basic Sciences, Faculty of Medicine, Universidad de La Frontera, Avenida Francisco Salazar 01145, Temuco 4811230, Chile

**Keywords:** maternal separation, adverse childhood experiences, postnatal stress, chronic stress, kidney, renal programming, kidney diseases, renal morphofunctionality

## Abstract

Exposure to stress during early developmental stages correlates with persistent alterations in multiple physiological systems, including the renal system. In rodents, maternal separation (MS) is a widely used experimental model to simulate postnatal adversity. Although this condition affects various renal parameters, a gap persists in knowledge regarding its impact on the functional unit of the kidney and the organization of the parenchyma. Thus, the objective of this systematic review was to analyze the effects of MS on the morphofunctional characteristics of the kidney in rodent models. We developed a protocol a priori following the SYRCLE and PRISMA guidelines and registered it in PROSPERO (CRD420251004703). We searched Web of Science, Scopus, Medline, Embase, BIREME-BVS, and SciELO without language or date restrictions, targeting experimental studies in rodents subjected to MS that evaluated structural, functional, or molecular alterations. Three independent reviewers performed data selection and extraction, and they assessed the risk of bias using the SYRCLE’s RoB tool. We included seven studies that met the eligibility criteria. At the structural level, studies reported cellular infiltrates positive for MPO, CD44, and TLR4, along with increased cortical and medullary microvascular density. Regarding renal function, the included studies described changes in ACE1 and ACE2 activity, oxidative stress, and enzymatic imbalance accompanied by a compensatory antioxidant response. At the molecular level, the studies reported variations in the expression of adrenergic receptors and the renin-angiotensin system. These findings suggest that MS may compromise the organization and functional integrity of the developing kidney, underscoring the need for studies that integrate structural and functional analyses in greater depth.

## 1. Introduction

Childhood represents a particularly sensitive stage of human development, during which physiological systems are undergoing maturation. During this period, exposure to adverse experiences, such as neglect, abuse, or loss of a primary caregiver, can lead to a state of chronic stress with long-term consequences on physical and mental health [1,2,3]. This condition, known as early developmental programming, has been widely studied and has been linked to a higher incidence of cardiovascular, metabolic, immunological, and renal diseases in adulthood [4,5,6,7,8].

In the experimental field, the maternal separation (MS) model in rodents represents a validated paradigm for investigating the effects of postnatal stress across multiple organs and physiological systems. This model involves repeatedly separating pups from their mother during the first postnatal days, a procedure that sustainably activates the hypothalamic–pituitary–adrenal axis and induces long-lasting neuroendocrine, immunological, and metabolic alterations [9,10,11]. Over the last few decades, evidence has shown that MS induces alterations in brain structure, behavior, emotional reactivity, and the physiology of peripheral organs, including the liver, heart, and kidney [12,13,14,15,16,17,18,19].

The kidney is a particularly vulnerable organ to early environmental influences, as its development extends into postnatal stages in many species, including rodents [20]. Renal organogenesis involves a complex sequence of events that includes the formation of nephrons, the maturation of the vascular network, and the establishment of functional autoregulatory mechanisms. External factors such as malnutrition, exposure to environmental chemicals, or stress can interfere with these processes, affecting morphogenesis and compromising the functional capacity of the kidney in later life stages [21,22].

In this sense, exposure to postnatal stress likely correlates with alterations in the renin–angiotensin system (RAS), endothelial dysfunction, changes in oxidative markers, and an inflammatory response in renal tissue [14,23]. However, despite the growing interest in this area, a significant gap remains in the literature regarding how postnatal stress specifically affects the functional unit of the kidney, which includes key structures such as the glomeruli, podocytes, basement membrane, fenestrated endothelial cells, proximal and distal tubules, and the interstitial compartment.

Furthermore, a comprehensive assessment of the effects of MS on the renal parenchyma must consider multiple levels of analysis from structural alterations visible by morphoanalytical and stereological techniques, to functional changes measured by biochemical tests, and molecular adjustments assessed by gene or protein expression. This multidimensional approach is indispensable for identifying early biomarkers of kidney damage and for proposing potential therapeutic targets [24].

Chronic kidney disease is now one of the leading causes of healthcare burden worldwide, and its incidence has increased significantly in recent decades. Current estimates indicate that more than 750 million people have some degree of kidney disease, and that by 2040, this condition will become the fifth leading cause of death worldwide [25,26]. In this scenario, identifying early risk factors and understanding the mechanisms that cause kidney damage becomes crucial for public health.

For all the above reasons, the present work aimed to carry out a systematic review of the available preclinical evidence on the effects of postnatal stress induced by MS on the structure and function of the kidneys in rodents. Through this review, we aim to integrate current knowledge, identify critical gaps in the literature, and contribute to understanding how early adversity can alter renal morphofunctionality and facilitate the development of pathologies later in life.

## 2. Materials and Methods

A systematic review was conducted, with its protocol developed a priori following the guidelines for systematic reviews of intervention studies in animals [27], and registered in the PROSPERO database under code CRD420251004703. This work follows the PRISMA (Preferred Reporting Items for Systematic Reviews and Meta-Analyses) statement [28].

### 2.1. Eligibility Criteria

Considering the acronym PICO, we included experimental studies in rodents (P). These studies assessed the impact of MS stress (I) on the morphofunctionality of the renal structure, along with modifications in tissue architecture as well as functional and molecular changes (O), regardless of the frequency or duration of the stressor. There were no restrictions on language or publication date. Systematic reviews, narratives, and letters to the editor that did not provide primary information were excluded, along with articles including rodents with existing pathologies or genetically modified rodents, as well as those that applied other interventions and did not independently report the results associated exclusively with MS.

### 2.2. Information Sources

We consulted the Web of Science (WoS), Scopus, Medline, and Embase databases, as well as virtual libraries such as BIREME-BVS and SciELO. Article searching and recruitment concluded on 6 March 2025. We conducted manual searches for cross-referenced articles and gray literature, using identified systematic reviews as an additional source of cross-references.

### 2.3. Search Strategy

We conducted sensitive searches for information, adapting the search strategy to each information source and utilizing MeSH, DeCS, and Emtree terms, as well as free terms combined with the Boolean operators “OR” and “AND” (Table 1). Two authors independently evaluated the search strategy, following the Peer Review of Electronic Search Strategies checklist [29].

### 2.4. Selection Process

We managed the documents identified in each information source using Rayyan software version 1.6.1 (Rayyan Systems Inc., Doha, Qatar), removing duplicates both automatically and manually. Two authors (JDC, JR) then screened the articles by title and abstract, applying the eligibility criteria. The same two authors, experienced in searching and analyzing biomedical studies, then retrieved the complete reports of potentially eligible studies and independently assessed each one. The reviewers resolved discrepancies at any stage by consensus; if necessary, they consulted a third reviewer (BV) to make a decision.

### 2.5. Data Collection Process

The data extraction process was performed independently by the three authors using a previously validated and standardized form. The reviewers resolved any inconsistencies by consensus. We collected the data in a database generated using Microsoft Excel 2019 software (Microsoft, Redmond, WA, USA).

### 2.6. Data Items

We divided the primary outcome variables into three categories: morphological, functional, and molecular, and reported them as continuous variables. We defined morphological alterations as quantifiable changes in the number, shape, size, arrangement, or volume of renal parenchymal structures, identified using morphometric, stereological, or other measurement methods. We defined functional changes as modifications in the activity, performance, or operational capacity of the kidney, measured through physiological and biochemical tests assessing parameters such as glomerular filtration, renal blood flow, and fluid and electrolyte balance. We defined molecular changes as alterations in gene or protein expression, identified using molecular biology and immunoassay tools, including PCR, microarrays, ELISA, or similar techniques.

### 2.7. Study Risk of Bias Assessment

Two researchers (JDC, JR) independently performed the risk of bias assessment using the SYRCLE RoB (Systematic Review Centre for Laboratory Animal Experimentation) tool for animal studies [30]. This tool contains 10 items that address six types of domains: selection bias, performance bias, detection bias, attrition bias, reporting bias, and other biases. Each entry was assessed based on the corresponding flagging questions: a “Yes” response reflected a low risk of bias, “No” corresponded to a high risk of bias, and “Unclear” indicated an unclear risk of bias due to insufficient information to make a definitive judgment. Responding “No” to any of the relevant signaling questions indicated a high risk of bias for that specific domain.

### 2.8. Effect Measures

Effect sizes were assessed using the mean difference as the measure of effect. This mean difference was calculated and analyzed in the meta-analysis using Meta-Plot software version 0.8.3 (StataCorp, College Station, TX, USA), allowing for a standardized comparison of results across the included studies.

### 2.9. Synthesis Methods

For effect measures, we used the standardized mean difference, which allows comparisons of results between studies that employed different scales to assess the outcomes of interest. As a synthesis method, a descriptive analysis of the results was applied, including measures of central tendency such as mean, median, and mode. In addition, we performed a meta-analysis when two or more studies reported comparable results, using a random-effects model and the Restricted Maximum Likelihood method. Heterogeneity was assessed by estimating Cochran’s Q, Tau^2^, I^2^, and 95% prediction intervals (95%PI).

### 2.10. Reporting Bias Assessment

Publication bias analysis was performed when ≥10 primary studies were included, funnel plot asymmetry was assessed, and Egger’s test was applied (*p* ≤ 0.05).

### 2.11. Certainty Assessment

We did not consider the use of certainty of evidence because this methodology establishes the degree of recommendation in clinical studies, which does not apply in the context of the present review.

## 3. Results

### 3.1. Study Selection

**Figure 1:**
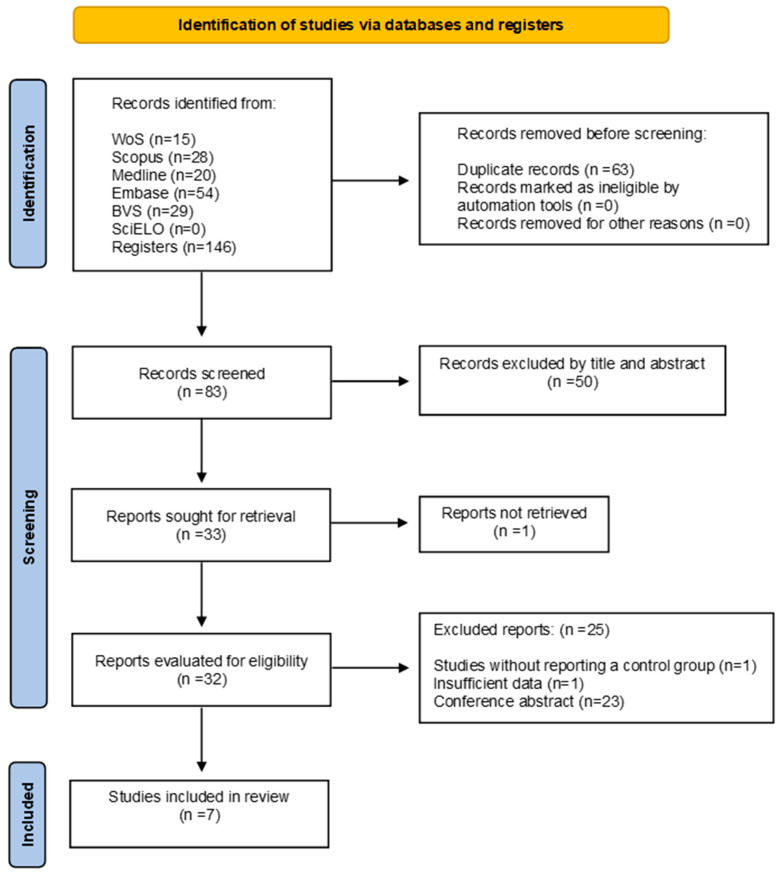
Flow diagram included studies.

We identified a total of 146 studies. Of these, we excluded 63 because they were duplicates. The remaining 83 articles underwent a title and abstract review, leading us to exclude 50 unrelated articles. Of the 33 articles reviewed in their entirety, one was unavailable, and we excluded 25 based on eligibility criteria. We did not identify any studies through cross-referencing, resulting in a total of 7 studies included in the final analysis [14,16,23,31,32,33,34] (Figure 1).

### 3.2. Study Characteristics

All included studies had a control group not exposed to MS or other interventions. In 57.14% (*n* = 4) of the studies, in addition to the control group and the group under-going MS, additional groups received other interventions. The format and duration of MS varied according to the animal model used, with differences observed between studies using rats and those using mice. Researchers conducted the studies in various animal models: 71.4% (*n* = 5) in Wistar Kyoto rats, 14.3% (*n* = 1) in C57BL/6 mice, and 14.3% (*n* = 1) in Sprague-Dawley rats. In 85.7% (*n* = 6) of cases, the experiments in-volved males, while in 14.3% (*n* = 1) the experiments involved both sexes. Table 2 summarizes these details.

### 3.3. Risk of Bias of Individual Studies

Table 3 shows the risk of bias assessment for each included study using SYRCLE’s RoB tool. Regarding the overall risk of bias, corresponding to the selection bias domain, 42.9% of the studies presented a moderate risk, while 57.1% showed a high risk. This assessment is mainly due to the lack of detailed information on random sequence generation and allocation concealment during the experimental protocol. Regarding performance bias, 57.1% of the studies showed a moderate risk and 42.9% a high risk, since in most cases the reports did not specify whether the animals had been randomized during housing or whether the researchers remained blinded to the interventions applied. Detection bias was present in a small proportion of low-risk studies (14.3%), which is related to the lack of information on blinding of evaluators and the lack of evidence on random selection of animals for outcome assessment. A similar issue occurred with attrition bias, where 85.7% of studies received a moderate level of concern, as the reports did not indicate how the researchers managed incomplete outcome data.

**Figure 2:**
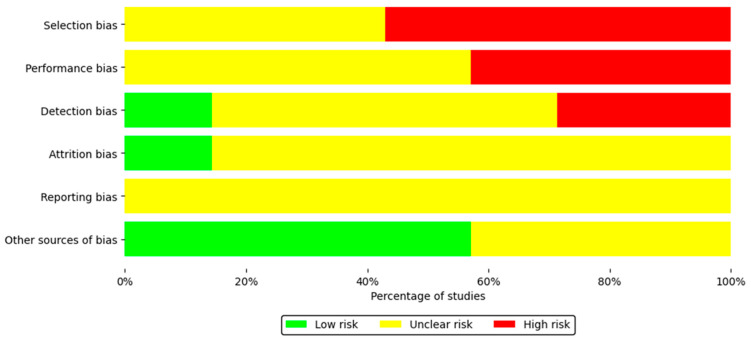
Summary of the risk of bias of the seven included studies.

Regarding reporting bias, this was assessed as a moderate risk because 100% of the studies lacked a prior accessible protocol; however, all clearly and consistently reported the prespecified primary and secondary outcomes. For other sources of bias, 57.1% of the studies were assessed as having low risk, and 42.9% were assessed as having moderate risk, as these studies did not report whether new animals were added to the control and experimental groups to replace those that had dropped out of the original population for various reasons (Figure 2).

### 3.4. Results of Syntheses

The selected studies evaluated the effects of MS stress on kidney structure and function in rodents. Researchers analyzed only the groups exposed to MS and their respective controls, excluding those that received additional interventions. Results were assessed using three types of analysis: morphoanalytical (stereological, morphometric, and analytical), functional, and molecular.

**Figure 3:**
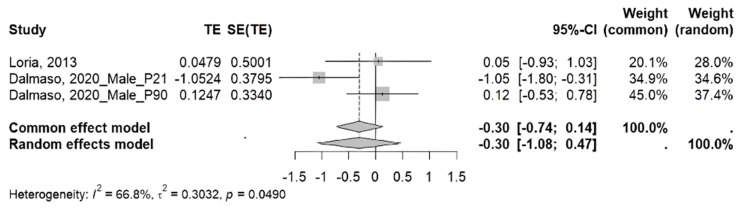
Meta-analysis of the effect of maternal separation on glomerular filtration rate in rats [23,31].

- **Morphoanalytic alterations:** Two articles analyzed the effects of MS on renal structure using morphoanalytic tests. De Miguel et al. [14] reported a significant increase in the proliferation of vasa recta endothelial cells in the outer medulla, as well as increased infiltration of MPO^+^ cells in the cortex and outer medulla of animals exposed to MS. Although other regions also showed increases, these differences were not significant. Additionally, a higher percentage of cortex and medulla stained positively for CD44 was reported, along with a marked increase in the rate of glomeruli containing CD44+ cells and in the average number of these cells per glomerulus. Finally, the number of TLR4+ cells in the outer medulla was significantly higher in the exposed group. For their part, Dalmasso et al. [23] indicated that, although renal mass at postnatal day 90 (P90) did not differ significantly, microvascular density increased markedly in the renal cortex and medulla of the MS group, both at P21 and P90, evaluated at depths of 0–200 µm and 0–500 µm. The details of this paragraph appear in Table 4.

- **Functional alterations:** Five studies evaluated the consequences of MS on renal function. Overall, parameters such as diuresis, natriuresis, renal blood flow, vascular resistance, renin, and aminopeptidase activity did not show significant differences [31,32,33]. However, significant alterations in the activity of enzymes of the renin-angiotensin system (ACE1 and ACE2) [23] and in antioxidant and oxidative stress markers were reported, with sex-dependent variations [16]. One of the studies employed a comparative approach, comprising four experimental groups: a stress group with MS from days P1–P15, P1–P14, and P15, and a control group without manipulation. This strategy enabled a differential evaluation of the impact of the type and intensity of separation on biochemical parameters, including the activities of superoxide dismutase, catalase, glutathione peroxidase, glutathione reductase, glutathione S-transferase, nitric oxide synthase, malondialdehyde, and ferric-reducing capacity [16]. The details of these findings appear in Table 5 and Table 6.

- **Molecular alterations:** Four studies evaluated the consequences of MS on renal function at the molecular level. Loria et al. [32] analyzed α1-adrenergic receptors and their gene expression, observing a significant decrease in the density of these receptors in the renal vasculature of the MS group, as well as an increase in the expression of the α1d-AR subtype in this same region. Cortical values and other receptor subtypes showed no significant differences. De Miguel et al. [14] reported a marked increase in IL-1β in the renal tissue of the MS group, with no changes in IL-4 and IL-6. For their part, Dalmasso et al. [23] described a significant decrease in lectin expression in the MS group, whereas the expression of perlecan, angiotensinogen mRNA, and other components of the renin-angiotensin system (such as MAS1 or neprilysin) did not show relevant variations. However, significant increases in the expression of the renin receptor and angiotensin type 1 and type 2 receptors, as well as aminopeptidase A, appeared at specific days of postnatal development. Conversely, ACE2 expression significantly decreased on days P6, P10, and P14 in the MS group. Likewise, angiotensin II and plasma renin concentrations changed, showing significant increases in the early stages. Finally, Mahanes et al. [33] reported elevated urinary angiotensinogen concentrations in the MS group, with no significant differences in mRNA or protein expression in the renal cortex. Table 7 presents the quantitative details of these findings.

- **Meta-analysis:** In a meta-analysis of studies evaluating glomerular filtration rate in rats, a trend toward increased glomerular filtration rate was observed in experimental groups compared to control groups. However, it did not reach statistical significance under the random effects model (ES = −0.30; 95% CI: −1.08 to 0.47; *p* > 0.05), nor under the common effects model (ES = −0.30; 95% CI: −0.74 to 0.14; *p* > 0.05). Likewise, the analysis revealed moderately high heterogeneity among the included studies (I^2^ = 66.8%; τ^2^ = 0.3032; *p* < 0.05) (Figure 3).

### 3.5. Reporting Biases

Due to the characteristics of the results, the proposed assessment of publication bias was not feasible.

## 4. Discussion

This is the first systematic review that integrated the available preclinical evidence on the effects of MS-induced postnatal stress on rodent renal structure. The retrieved studies provided information on how this type of stress affects the kidney in various ways: at the morphological level, reflecting changes in tissue and cellular architecture; at the functional level, revealing alterations in enzymatic activity and redox balance; and at the molecular level, revealing changes in the expression and regulation of receptors, inflammatory mediators, and elements of the renin-angiotensin system. These integrated effects are described and discussed below.

### 4.1. Morphological Impact

MS caused an increase in MPO^+^, CD44^+^ cells in the glomeruli and TLR4^+^ cells in the kidneys of rats [14], suggesting an activation of the renal inflammatory environment. These markers are associated with activated infiltrating or resident immune cells that participate in the local inflammatory response. This finding is consistent with that reported by Ha et al. [35], who suggest that early stress induces persistent systemic inflammation with direct effects on renal tissue. In this context, immune activation promotes the release of cytokines and chemokines, which act as mediators not only amplifying inflammation but also stimulating cell proliferation in the renal parenchyma [36], potentially explaining the increased proliferation of endothelial cells in the vasa recta of the outer medulla [14]. This is closely related to the increased microvascular density in the kidney of rats exposed to MS [23], since cytokines and chemokines involved in inflammation contribute to microvascular changes by acting synergistically [37]; in particular, CXCL6 and CCL2 enhance neutrophil chemotaxis, which indirectly favors angiogenesis through increased leukocyte infiltration and the subsequent release of proangiogenic factors [38]. While initial increases in microvascular density may serve as a compensatory response, over time they can lead to microvascular rarefaction, thereby exacerbating renal injury and fibrosis [39], which are common pathways for the development of chronic kidney disease [40].

Despite current advances, a notable lack of studies still exists that specifically analyze the effect of MS on critical structural components of the kidney’s functional unit, such as glomeruli, podocytes, the glomerular basement membrane, and fenestrated endothelial cells. These structures constitute the glomerular filtration barrier, a highly specialized multicellular complex that regulates the selective passage of water and solutes from the blood into the urinary tract, thereby maintaining homeostasis and preventing plasma protein loss. Its study allows the identification of early morphological alterations associated with glomerular filtration dysfunction, such as proteinuria or loss of vascular integrity. This approach, focused on the renal corpuscle, can be complemented by the analysis of other structures equally relevant to the overall function of the organ, such as the renal tubules, responsible for reabsorption, secretion, and fluid and electrolyte balance, as well as elements of the interstitium and vascular compartment, whose alterations may reflect inflammatory processes, fibrosis, or hemodynamic damage. Taken together, this comprehensive approach would enable a more precise characterization of the impact of early stress on renal parenchymal architecture and function.

### 4.2. Functional Impact

MS-induced alterations in the activity of various enzymes, with a particular increase observed in ACE1 and ACE2 activity [23]. This finding suggests a stress-induced activation of the renin-angiotensin system, primarily mediated by the upregulation of ACE1, which promotes the production of angiotensin II peptide with vasoconstrictor, pro-inflammatory, and oxidative stress-inducing effects that may contribute to the functional deterioration of renal tissue [41,42]. For its part, the increase in ACE2 activity could be interpreted as an adaptive response to excess angiotensin II, since this enzyme promotes the formation of angiotensin 1-7, a peptide with vasodilatory and anti-inflammatory effects [43].

**Figure 4:**
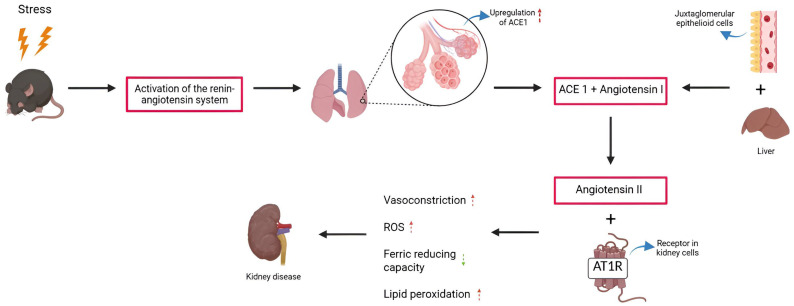
Imbalance of the renin-angiotensin system and oxidative stress in the kidneys of rodents exposed to maternal separation. Red arrows indicate increase, green arrows decrease.

The imbalance between the enzymes of the renin-angiotensin system reflects an alteration associated with stress induced by MS, generating an environment conducive to kidney damage, partially mediated by oxidative stress. This condition favors the increase in reactive oxygen species (ROS), which leads to a reduction in ferric-reducing capacity and induces lipid peroxidation, as evidenced by the elevated malondialdehyde levels—an observation consistent with the results reported by Fenton-Navarro et al. [16] in the kidneys of rats exposed to chronic stress due to MS (Figure 4). Thus, elevated levels of ROS and malondialdehyde, together with low levels of ferric-reducing capacity, are consistently associated with kidney damage and, consequently, with the progression of kidney diseases [44,45].

Faced with this redox imbalance, the observed increase in the activity of antioxidant enzymes, including superoxide dismutase, catalase, glutathione peroxidase, and glutathione reductase, which neutralize ROS, likely reflects an adaptive response to this condition. However, this response would not be completely effective, as reflected by the expression of nitric oxide synthase in the groups not subjected to MS, whose function would be oriented to preserving vascular integrity and mitigating the impact of the pro-oxidant environment [16].

Regarding the meta-analysis of glomerular filtration rate, the included studies report divergent results, with both increases and decreases observed after MS. Overall, the analysis revealed an increase in glomerular filtration rate, although this increase was not significant. In addition, considerable heterogeneity is evident (I^2^ = 66.8%), despite the included studies employing the same animal model and MS format. Perhaps these differences are attributable to the age at which the rats underwent assessment, since Loria et al. [31] measured this parameter between P84 and P98, while Dalmasso et al. [23] assessed it twice, on P21 and P90. Taken together, these findings do not provide a conclusive effect of MS on glomerular filtration rate in rodents, highlighting the need for further studies that elucidate how this parameter is affected by chronic MS stress.

### 4.3. Molecular Impact

The renal vasculature of rats exposed to chronic MS stress shows a reduced density of α_1_-adrenergic receptors (AR), particularly of the α_1_d-AR subtype [31]. This decrease could result from a desensitization process secondary to chronic overstimulation of renal sympathetic flow, a mechanism mediated by receptor phosphorylation that interferes with receptor function and drives receptor internalization [46]. Under physiological conditions, a lower density of these receptors could lead to an attenuated vasoconstrictor response and, consequently, to blood pressure within normal or reduced ranges. However, decreased α_1_d-AR function under chronic stress conditions can lead to a compensatory increase in other adrenergic receptors, such as β-adrenergic receptors (β-ARs), whose chronic activation is part of a vicious cycle that exacerbates heart failure [47]. In turn, chronic overactivation of these receptors promotes systemic inflammation [48], a process in which IL-1β functions as a key mediator.

In this context, the increased renal IL-1β expression observed in rats exposed to MS [14] could contribute to the establishment of a proinflammatory environment in renal tissue, creating a proinflammatory state that favors dysregulation of the microvasculature at this level [37]. This dysregulation is reflected in the marked reduction in tomato lectin staining in the vascular endothelium, indicating alterations in these cells as a consequence of MS stress [23].

The imbalance in the renal microvasculature, specifically an increase in its density induced by MS, could result from this type of stress deregulating glucocorticoid levels, a hormone known to regulate elements of the RAS [49]. During the first postnatal days, MS induces an increase in the mRNA expression of key RAS receptors, including the renin receptor, angiotensin type I and II receptors, aminopeptidase A, and plasma renin. However, from day P21 onwards, these levels tend to normalize [23], likely due to compensatory post-transcriptional mechanisms, because lncRNAs interact with microRNAs, inhibiting their activity and directly affecting the stability and translation of mRNAs [50]. This interaction could contribute to the suppression of RAS gene expression, indicating an adaptive response of the organism to prolonged stress aimed at preserving functional homeostasis. This compensatory mechanism was also evident starting at P21 for angiotensin II; however, unlike the other RAS components, angiotensin II decreased in rats exposed to MS [23]. This decrease is due to periods of reduced ACE2 mRNA expression. Initially, angiotensin II upregulates ACE2, but over time, it induces downregulation through receptor internalization and lysosomal degradation, a process mediated by the angiotensin II type 1 receptor [51].

### 4.4. Epigenetic Pathways

Although MS caused alterations at the morphological, functional, and molecular levels, with consequences summarized in Table 8, these effects appear to be mediated, at least in part, by epigenetic mechanisms that stably modulate gene expression without altering the DNA sequence. Among these mechanisms, hypermethylation of the RASA1 gene stands out, associated with renal fibrosis and deterioration of renal function [52]. Likewise, hypermethylation of the NR3C1 promoter has been observed in response to stress during early life stages, leading to altered expression of the glucocorticoid receptor [53], a change linked to the development of hypertension, diabetic nephropathy, and chronic kidney disease [54]. Despite these findings, the available literature on epigenetic pathways altered by early stress due to maternal separation and its specific impact on renal structure and function remains limited. Therefore, advancing studies that integrate epigenomic, transcriptional, and functional analyses is essential to elucidate the mechanisms involved and to identify therapeutic targets capable of mitigating early stress-induced kidney damage.

### 4.5. Limitations

Overall, the included studies showed a moderate to high risk of bias, mainly linked to selection and detection bias, which resulted from the limited reporting of randomization and allocation concealment procedures. Furthermore, few studies adequately addressed the handling of incomplete data or mentioned the inclusion of replacement animals, which compromises the internal validity of the results. An additional relevant limitation is sex bias, since only one of the seven studies analyzed included both males and females, while the others used only male animals, making it impossible to generalize the findings to both sexes. On the other hand, regarding the glomerular filtration rate, only three studies met the inclusion criteria for the meta-analysis, which limits the statistical robustness of the results obtained for this functional parameter. Finally, it should be acknowledged that four of the seven included studies were conducted by the same research groups, which may introduce an additional source of bias and affect the generalizability of the findings. This lack of diversity among research sources could also influence the homogeneity of the results and limit the external validity of the meta-analysis; therefore, the findings should be interpreted with caution. Further independent studies are needed to confirm and expand these observations.

## 5. Conclusions

MS is a well-established experimental model for studying the effects of postnatal stress on various physiological systems, including the renal system. The evidence gathered in this systematic review indicates that MS can generate significant structural, functional, and molecular alterations in the rodent kidney, characterized by inflammatory infiltrates, oxidative stress, RAS activation, and changes in vascular and enzyme density. These findings support the hypothesis that early adversity can lead to the negative programming of kidney development, with potential long-term health implications.

However, the available studies present a high degree of heterogeneity in terms of experimental design, age of assessment, techniques used, and variables analyzed, which makes quantitative integration of the results difficult. Furthermore, research on key elements of the renal functional unit, including glomeruli, podocytes, fenestrated endothelial cells, renal tubules, and the interstitial compartment, remains limited, particularly when applying detailed morphological methodologies and comparable quantitative analyses.

In this context, new research is required that integrates histological, quantitative, and molecular approaches to achieve a more accurate understanding of the impact of postnatal stress on renal architecture and function. Such knowledge will allow us not only to identify early biomarkers of damage but also to advance strategies for the prevention and treatment of chronic kidney diseases associated with early-onset conditions.

## Figures and Tables

**Table 1:** Search strategies.

Metasearch Engines and Databases	Search Strategies
WoS	(TS = (Rodentia OR Mice OR Rats OR Rodentia * OR Rat OR Rattus OR Rodent OR Mus OR Mouse OR Murine OR “Mus musculus”)) AND (TS = (“Maternal separation” OR “Maternal deprivation” OR “Deprivation maternal” OR “Neonatal stress” OR “Postnatal stress” OR “Early life stress” OR “Life stress early” OR “Stress early life”)) AND (TS = (Kidney OR Diuresis OR Diureses OR “Glomerular filtration rates” OR “Filtration rate glomerular” OR “Effective renal plasma flow” OR “Renal plasma flow, effective” OR ERPF OR “Renal blood flow effective” OR “Renal blood flow, effective” OR “Effective renal blood flow” OR ERBF OR “Cortex kidney” OR “Glomerular filtration barrier” OR “Glomerular basement membrane *” OR Podocyte OR “Glomerular visceral epithelial cells” OR “Juxtaglomerular apparatus” OR “Mesangial cell *” OR “Kidney mesangial cell *” OR “Renal mesangial cells”)))
Scopus	(TITLE-ABS-KEY (rodentia OR mice OR rats OR rodentia * OR rat OR rattus OR rodent OR mus OR mouse OR murine OR “Mus musculus”)) AND (TITLE-ABS-KEY (“Maternal separation” OR “Maternal deprivation” OR “Deprivation maternal” OR “Neonatal stress” OR “Postnatal stress” OR “Early life stress” OR “Life stress early” OR “Stress early life”)) AND (TITLE-ABS-KEY (kidney OR diuresis OR diureses OR “Glomerular filtration rates” OR “Filtration rate glomerular” OR “Effective renal plasma flow” OR erpf OR “Renal blood flow effective” OR “Effective renal blood flow” OR erbf OR “Cortex kidney” OR “Glomerular filtration barrier” OR “Glomerular basement membrane *” OR podocyte OR “Glomerular visceral epithelial cells” OR “Juxtaglomerular apparatus” OR “Mesangial cell *” OR “Kidney mesangial cell *” OR “Renal mesangial cells”))
Medline	(“Rodentia” [MeSH Terms] OR “Mice” [MeSH Terms] OR “Rats” [MeSH Terms] OR “rodentia *” [Title/Abstract] OR “Mice” [Title/Abstract] OR “Rat” [Title/Abstract] OR “Rats” [Title/Abstract] OR “Rattus” [Title/Abstract] OR “rodent *” [Title/Abstract] OR “Mus” [Title/Abstract] OR “Mouse” [Title/Abstract] OR “Murine” [Title/Abstract] OR “Mus musculus” [Title/Abstract] OR ((“Rat” [Title/Abstract] OR “Rats” [Title/Abstract] OR “Mouse” [Title/Abstract]) AND “Laboratory” [Title/Abstract])) AND (“Rodentia” [MeSH Terms] OR “Mice” [MeSH Terms] OR “Rats” [MeSH Terms] OR “rodentia *” [Title/Abstract] OR “Mice” [Title/Abstract] OR “Rat” [Title/Abstract] OR “Rats” [Title/Abstract] OR “Rattus” [Title/Abstract] OR “rodent *” [Title/Abstract] OR “Mus” [Title/Abstract] OR “Mouse” [Title/Abstract] OR “Murine” [Title/Abstract] OR “Mus musculus” [Title/Abstract] OR ((“Rat” [Title/Abstract] OR “Rats” [Title/Abstract] OR “Mouse” [Title/Abstract]) AND “Laboratory” [Title/Abstract])) AND (“Maternal Deprivation” [MeSH Terms] OR “Maternal separation” [Title/Abstract] OR “maternal deprivation *” [Title/Abstract] OR “deprivation maternal” [Title/Abstract] OR “neonatal stress *” [Title/Abstract] OR “postnatal stress *” [Title/Abstract] OR “early life stress *” [Title/Abstract] OR “life stress early” [Title/Abstract] OR “stress early life” [Title/Abstract]) AND (“Kidney” [MeSH Terms] OR “kidney *” [Title/Abstract] OR “Diuresis” [MeSH Terms] OR “Diuresis” [Title/Abstract] OR “Diureses” [Title/Abstract] OR “Glomerular Filtration Rate” [MeSH Terms] OR “Glomerular Filtration Rates” [Title/Abstract] OR “filtration rate glomerular” [Title/Abstract] OR “renal plasma flow, effective” [MeSH Terms] OR “Effective Renal Plasma Flow” [Title/Abstract] OR “ERPF” [Title/Abstract] OR “renal blood flow, effective” [MeSH Terms] OR “renal blood flow effective” [Title/Abstract] OR “Effective Renal Blood Flow” [Title/Abstract] OR “ERBF” [Title/Abstract] OR “cortex kidney” [Title/Abstract] OR “Glomerular Filtration Barrier” [Title/Abstract] OR “glomerular basement membrane *” [Title/Abstract] OR “Kidney Glomerulus” [MeSH Terms] OR “Podocytes” [MeSH Terms] OR “podocyte *” [Title/Abstract] OR “Glomerular Visceral Epithelial Cells” [Title/Abstract] OR “Juxtaglomerular Apparatus” [Title/Abstract] OR “mesangial cell *” [Title/Abstract] OR “kidney mesangial cell *” [Title/Abstract] OR “Renal Mesangial Cells” [Title/Abstract])
Embase	(‘rodent’/exp OR ‘rodent’ OR ‘mouse’/exp OR ‘mouse’ OR ‘rat’/exp OR ‘rat’ OR ‘mus musculus’/exp OR ‘mus musculus’ OR ‘murine’/exp OR ‘murine’ OR ‘rodentia’: ti, ab OR ‘mice’: ti, ab OR ‘rats’: ti, ab OR ‘rattus’: ti, ab) AND (‘maternal deprivation’/exp OR ‘maternal deprivation’ OR ‘neonatal stress’/exp OR ‘neonatal stress’ OR ‘postnatal stress’/exp OR ‘postnatal stress’ OR ‘early life stress’/exp OR ‘early life stress’ OR ‘maternal separation’: ti, ab OR ‘deprivation materna’: ti, ab OR ‘life stress early’: ti, ab OR ‘stress early life’: ti, ab) AND (‘kidney’/exp OR ‘kidney’ OR ‘diuresis’/exp OR ‘diuresis’ OR ‘glomerulus filtration rate’/exp OR ‘glomerulus filtration rate’ OR ‘effective kidney plasma flow’/exp OR ‘effective kidney plasma flow’ OR ‘kidney blood flow’/exp OR ‘kidney blood flow’ OR ‘podocyte’/exp OR ‘podocyte’ OR ‘kidney cortex’/exp OR ‘kidney cortex’ OR ‘glomerular filtration barrier’/exp OR ‘glomerular filtration barrier’ OR ‘glomerulus basement membrane’/exp OR ‘glomerulus basement membrane’ OR ‘juxtaglomerular apparatus’/exp OR ‘juxtaglomerular apparatus’ OR ‘mesangium cell’/exp OR ‘mesangium cell’ OR ‘rmc cell line (kidney)’/exp OR ‘rmc cell line (kidney)’ OR ‘kidney glomerulus’: ti, ab OR ‘diureses’: ti, ab OR ‘filtration rate glomerular’: ti, ab OR ‘erpf’: ti, ab OR ‘erbf’: ti, ab)
BVS	(“Rodentia *” OR “Mice” OR “Rats” OR “Rat” OR “Rodent *” OR “Rattus” OR “Mus” OR “Mouse” OR “Murine” OR “Roedores” OR “Ratones” OR “Ratón” OR “Rata” OR “Ratas” OR “Murinae” OR “Camundongos” OR “Camundongo” OR “Ratos” OR “Rato”) AND (“maternal deprivation” OR “privación materna” OR “privação materna” OR “séparation d’avec la mère” OR “maternal separation” OR “neonatal stress” OR “postnatal stress” OR “adverse childhood experiences” OR “experiencias adversas de la infancia” OR “experiências adversas da infância” OR “expériences défavorables de l’enfance” OR “early life stress” OR “estrés de la vida temprana” OR “Life stress early” OR “Stress early life”) AND (“kidney” OR “riñón” OR “rim” OR “rein” OR “diuresis” OR “diurese” OR “diurèse” OR “glomerular filtration rate” OR “Tasa de Filtración Glomerular” OR “Taxa de Filtração Glomerular” OR “Débit de filtration glomérulaire” OR “Renal Plasma Flow, Effective” OR “Flujo Plasmático Renal Efectivo” OR “Fluxo Plasmático Renal Efetivo” OR “Débit plasmatique rénal efficace” OR “EFPR” OR “Renal Blood Flow, Effective” OR “Flujo Sanguíneo Renal Efectivo” OR “Fluxo Sanguíneo Renal Efetivo” OR “Débit sanguin rénal efficace” OR “ERBF” OR “Kidney Cortex” OR “Corteza Renal” OR “Córtex Renal” OR “Cortex rénal” OR “Glomerular filtration barrier” OR “Barrera de Filtración Glomerular” OR “Barreira de Filtração Glomerular” OR “Barrière de filtration glomérulaire” OR “Glomerular basement membrane *” OR “Membrana Basal Glomerular” OR “Membrane basale glomérulaire” OR “Podocytes” OR “Podocitos” OR “Glomerular visceral epithelial cells” OR “Juxtaglomerular apparatus” OR “Aparato Yuxtaglomerular” OR “Sistema Justaglomerular” OR “Appareil juxtaglomérulaire” OR “Mesangial cell *” OR “Células Mesangiales” OR “Células Mesangiais”) AND instance:“regional”
SciELO	(“Rodentia *” OR “Mice” OR “Rats” OR “Rat” OR “Rodent *” OR “Rattus” OR “Mus” OR “Mouse” OR “Murine” OR “Roedores” OR “Ratones” OR “Ratón” OR “Rata” OR “Ratas” OR “Murinae” OR “Camundongos” OR “Camundongo” OR “Ratos” OR “Rato”) AND (“maternal deprivation” OR “privación materna” OR “privação materna” OR “sépara-tion d’avec la mère” OR “maternal separation” OR “neonatal stress” OR “postnatal stress” OR “adverse childhood experiences” OR “experiencias adversas de la infancia” OR “experiências adversas da infância” OR “expériences défavorables de l’enfance” OR “early life stress” OR “estrés de la vida temprana” OR “Life stress early” OR “Stress early life”) AND (“kidney” OR “riñón” OR “rim” OR “rein” OR “diuresis” OR “diurese” OR “di-urèse” OR “glomerular filtration rate” OR “Tasa de Filtración Glomerular” OR “Taxa de Filtração Glomerular” OR “Débit de filtration glomérulaire” OR “Renal Plasma Flow, Effective” OR “Flujo Plasmático Renal Efectivo” OR “Fluxo Plasmático Renal Efetivo” OR “Débit plasmatique rénal efficace” OR “EFPR” OR “Renal Blood Flow, Effective” OR “Flujo Sanguíneo Renal Efectivo” OR “Fluxo Sanguíneo Renal Efetivo” OR “Débit san-guin rénal efficace” OR “ERBF” OR “Kidney Cortex” OR “Corteza Renal” OR “Córtex Renal” OR “Cortex rénal” OR “Glomerular filtration barrier” OR “Barrera de Filtración Glomerular” OR “Barreira de Filtração Glomerular” OR “Barrière de filtration glomé-rulaire” OR “Glomerular basement membrane *” OR “Membrana Basal Glomerular” OR “Membrane basale glomérulaire” OR “Podocytes” OR “Podocitos” OR “Glomerular vis-ceral epithelial cells” OR “Juxtaglomerular apparatus” OR “Aparato Yuxtaglomerular” OR “Sistema Justaglomerular” OR “Appareil juxtaglomérulaire” OR “Mesangial cell *” OR “Células Mesangiales” OR “Células Mesangiais”)

The asterisk (*) indicates the use of the truncation operator in the search strategies.

**Table 2:** Characteristics of the interventions.

Authors	Groups	Additional Intervention	Maternal Separation Format (Postnatal Day)	Duration of Maternal Separation (h)	Animal Model	Sex	Number of Animals Per Group
Loria et al., 2013 [31]	CRD; CWRD; MSWRD; MSRD	Renal denervation	P2–P14	3	Wistar Kyoto rats	Males	6
Loria et al., 2017 [32]	C; MS	Without intervention	P2–P14	3	Wistar Kyoto rats	Males	4–8
De Miguel et al., 2018 [14]	C; MS	Without intervention	P2–P14	3	Wistar Kyoto rats	Males	4–8
Dalmasso et al., 2020 [23]	C; MS	Without intervention	P2–P14	3	Wistar Kyoto rats	Males	5–8
Mahanes et al., 2020 [33]	CCD; CHF; MSCD; MSHF	High-fat diet	P2–P16	3	Wistar Kyoto rats	Males	6–9
Dalmasso et al., 2024 [34]	CCD; CHF; MSCD; MSHF	High-fat diet	P2–P17	4 (P2–P5) and 8 (P6–P16)	C57BL/6 mice	Males	6
Fenton-Navarro et al., 2024 [16]	C; MS	Acute maternal separation stress	P15; P1–P14; P1–P15	3	Sprague-Dawley rats	Males and females	8–10

C: Control; MS: Maternal separation; CWRD: Control without renal denervation; CRD: Control with renal denervation; MSWRD: Maternal separation without renal denervation; MSRD: Maternal separation with renal denervation; CCD: Control with control diet; CHF: Control with high-fat diet; MSCD: Maternal separation with control diet; MSHF: Maternal separation with high-fat diet; P: (posnatal day).

**Table 3:** Risk of bias of included studies according to the SYRCLE RoB tool.

Authors	Selection Bias	Performance Bias	Detection Bias	Attrition Bias	Reporting Bias	Other Sources of Bias
Loria et al., 2013 [31]	High risk	Unclear risk	Unclear risk	Unclear risk	Unclear risk	Unclear risk
Loria et al., 2017 [32]	High risk	Unclear risk	Unclear risk	Unclear risk	Unclear risk	Unclear risk
De Miguel et al., 2018 [14]	Unclear risk	Unclear risk	High risk	Unclear risk	Unclear risk	Low risk
Dalmasso et al., 2020 [23]	Unclear risk	Unclear risk	Low risk	Unclear risk	Unclear risk	Low risk
Mahanes et al., 2020 [33]	High risk	High risk	Unclear risk	Low risk	Unclear risk	Low risk
Dalmasso et al., 2024 [34]	High risk	High risk	Unclear risk	Unclear risk	Unclear risk	Unclear risk
Fenton-Navarro et al., 2024 [16]	Unclear risk	High risk	High risk	Unclear risk	Unclear risk	Low risk

**Table 4:** Effects of maternal separation on kidney morphology in rodents.

Authors	Parameter	Region/Time	MS (Mean ± SE)	Control (Mean ± SE)	*p*-Value
De Miguel et al., 2018 [14]	Cell proliferation (cells/field)	Outer Medulla	12.37 ± 2.07	4.91 ± 0.80	<0.05
		Cortex	10.31 ± 1.45	5.72 ± 0.80	>0.05
		Inner Medulla	7.89 ± 2.68	8.18 ± 0.61	>0.05
	MPO^+^ cells (cells/field)	Outer Medulla	2.80 ± 0.26	1.67 ± 0.37	<0.05
		Cortex	2.09 ± 0.14	1.26 ± 0.18	<0.05
		Inner Medulla	0.77 ± 0.23	0.62 ± 0.25	>0.05
	CD44^+^ stained area (%)	Cortex	3.83 ± 0.83	2.81 ± 1.80	>0.05
		Medulla	1.34 ± 0.37	0.54 ± 0.13	>0.05
	CD44^+^ in glomeruli (%)	Glomeruli	67.60 ± 4.49	49.80 ± 4.13	<0.05
	(cells/glomerulus)	Glomerulus	1.29 ± 0.16	0.79 ± 0.12	<0.05
	TLR4^+^ cells (cells/field)	Outer Medulla	2.92 ± 0.22	1.50 ± 0.28	<0.05
		Inner Medulla	1.07 ± 0.22	1.11 ± 0.18	>0.05
Dalmasso et al., 2020 [23]	Kidney mass (g)	P90	1.88 ± 0.09	1.82 ± 0.0	>0.05
	Microvascular density (MV/mm^2^)	Medulla 0–200 µm/P21	313.04 ± 47.83	203.86 ± 37.59	<0.05
		Medulla 0–500 µm/P21	352.17 ± 46.38	241.45 ± 33.25	<0.05
		Cortex 0–200 µm/P21	424.64 ± 53.62	275.36 ± 52.18	<0.05
		Cortex 0–500 µm /P21	478.85 ± 51.92	350.48 ± 53.37	<0.05
		Medulla 0–200 µm/P90	237.42 ± 33.13	145.40 ± 23.93	<0.05
		Medulla 0–500 µm/P90	267.89 ± 23.93	165.14 ± 25.69	<0.05
		Cortex 0–200 µm/P90	312.67 ± 51.16	192.07 ± 18.30	<0.05
		Cortex 0–500 µm/P90	362.20 ± 53.04	225.00 ± 14.63	<0.05

MS: Maternal separation; SE: Standard error.

**Table 5:** Effects of maternal separation on renal function in rodents.

Authors	Sex	Parameter	MS (Mean ± SE)	Control (Mean ± SE)	*p*-Value
Loria et al., 2013 [31]	Males	Diuresis (mL/day)	26.1 ± 2.2	27.3 ± 3.4	>0.05
		Natriuresis (mEq/day)	1.73 ± 0.11	1.67 ± 0.06 vs	>0.05
Loria et al., 2017 [32]	Males	Renal blood flow (mL/min)	12.06 ± 0.59	11.88 ± 0.86	>0.05
		Renal vascular resistance (mmHg·min/mL)	6.61 ± 0.44	6.83 ± 0.44	>0.05
Dalmasso et al., 2020 [23]	Males	ACE1 (RFU/min/µg protein)	62.45 ± 7.59	30.80 ± 3.80	<0.05
		ACE2 (RFU/min/µg protein)	168.64 ± 8.90	58.47 ± 16.95	<0.05
Dalmasso et al., 2024 [34]	Males	Renin activity (pg/mL)	1312.98 ± 580.92	793.89 ± 274.81	>0.05
		Aminopeptidase A (pg/mL)	0.08 ± 0.02	0.10 ± 0.03	>0.05

MS: Maternal separation; SE: Standard error.

**Table 6:** Effects of maternal separation on renal function in rodents [16].

Parameter	Sex	MS P1–P15 (Mean ± SE)	MS P1–P14 (Mean ± SE)	MS P15 (Mean ± SE)	Control (Mean ± SE)	*p*-Value
Superoxide dismutase (U/mg prot)	Male	4.86 ± 0.05 ^a,b,c^	5.26 ± 0.11 ^a,b^	5.66 ± 0.45 ^a^	3.26 ± 0.17	<0.05
	Female	5.58 ± 0.06 ^a,b,c^	5.09 ± 0.06 ^a^	5.15 ± 0.24 ^a^	3.58 ± 0.18	<0.05
Catalase (U/mg prot)	Male	21.94 ± 0.92 ^a,b,c^	41.60 ± 4.11 ^b^	32.91 ± 3.66 ^a^	42.51 ± 2.29	<0.05
	Female	25.76 ± 1.81 ^a,b,c^	44.75 ± 1.35 ^a,b^	33.45 ± 1.35 ^a^	38.87 ± 3.27	<0.05
Glutathione peroxidase (U/mg prot)	Male	0.70 ± 0.06 ^a,b,c^	0.27 ± 0.03	0.34 ± 0.08	0.28 ± 0.08	<0.05
	Female	0.76 ± 0.11 ^a,b,c^	0.23 ± 0.02	0.28 ± 0.09	0.26 ± 0.10	<0.05
Glutathione reductase (μmol/mg prot)	Male	0.40 ± 0.03	0.79 ± 0.01 ^a,b^	0.41 ± 0.04	0.41 ± 0.03	<0.05
	Female	0.54 ± 0.04 ^c^	0.68 ± 0.11 ^a,b^	0.52 ± 0.01	0.49 ± 0.05	<0.05
Glutathione S-transferase (U/mL)	Male	0.015 ± 0.002 ^a,b^	0.018 ± 0.003	0.020 ± 0.05	0.023 ± 0.003	<0.05
	Female	0.014 ± 0.002 ^a^	0.015 ± 0.004 ^a^	0.017 ± 0.004 ^a^	0.025 ± 0.004	<0.05
Nitric oxide synthase (μmol/mg)	Male	0.76 ± 0.12 ^a,b,c^	0.99 ± 0.09 ^a^	1.00 ± 0.14 ^a^	1.71 ± 0.17	<0.05
	Female	0.76 ± 0.13 ^a,b^	0.87 ± 0.17 ^a,b^	1.30 ± 0.16	1.25 ± 0.26	<0.05
Malondialdehyde (nmol/g)	Male	89.61 ± 5.84 ^a,b,c^	69.16 ± 2.92 ^a,b^	38.96 ± 9.74 ^a^	26.30 ± 1.95	<0.05
	Female	95.64 ± 6.04 ^a,b,c^	62.42 ± 9.06 ^a,b^	41.28 ± 5.03 ^a^	29.19 ± 3.02	<0.05
Ferric-reducing capacity (μmol/mg FeSO_4_)	Male	439.31 ± 18.49 ^a,b,c^	564.16 ± 13.87 ^a^	541.04 ± 50.87 ^a^	628.90 ± 23.12	<0.05
	Female	593.87 ± 19.63 ^a^	584.05 ± 14.72 ^a^	579.14 ± 49.08 ^a^	539.88 ± 30.54	<0.05

MS: Maternal separation; P: postnatal day; SE: Standard error. ^a^ difference with the control group; ^b^ difference with the MS P15 group; ^c^ differences with the MS P1–P14 group.

**Table 7:** Effects of maternal separation on molecular parameters in the rodent kidney.

Authors	Parameter	MS (Mean ± SE)	Control (Mean ± SE)	*p*-Value
Loria et al., 2013 [31]	α_1_-AR cortical tissue (fmol/mg protein)	41.69 ± 4.85	43.46 ± 1.19	>0.05
	α_1_-AR renal vasculature (fmol/mg protein)	46.48 ± 4.4	64.96 ± 69.76	<0.05
	α_1_a-AR mRNA renal cortex (2^−ΔΔCt^)	1.68 ± 0.26	1.19 ± 0.29	>0.05
	α_1_b-AR mRNA renal cortex (2^−ΔΔCt^)	1.40 ± 0.27	0.93 ± 0.29	>0.05
	α_1_d-AR mRNA renal cortex (2^−ΔΔCt^)	1.29 ± 0.14	0.99 ± 0.05	>0.05
	α_1_a-AR mRNA renal vasculature (2^−ΔΔCt^)	1.17 ± 0.16	1.11 ± 0.19	>0.05
	α_1_b-AR mRNA renal vasculature (2^−ΔΔCt^)	0.63 ± 0.10	0.56 ± 0.09	>0.05
	α_1_d-AR mRNA renal vasculature (2^−ΔΔCt^)	0.19 ± 0.05	0.03 ± 0.01	<0.05
De Miguel et al., 2018 [14]	Renal IL-1β (pg/mg)	7.91 ± 1.02	4.44 ± 0.48	<0.05
	Renal IL-4 (pg/mg)	4.52 ± 1.04	3.98 ± 1.87	>0.05
	Renal IL-6 (pg/mg)	6.71 ± 0.65	5.96 ± 1.10	>0.05
Dalmasso et al., 2020 [23]	Perlecan (perlecan/GAPDH)	0.90 ± 0.20	1.10 ± 0.10	>0.05
	Lectin (PP)	32.687 ± 4.185	52.578 ± 5.988	<0.05
	mRNA angiotensinogen	P2: 23.47 ± 10.39	P2: 5.64 ± 0.89	>0.05
		P6: 19.60 ± 2.68	P6: 3.56 ± 2.68	>0.05
		P10: 27.92 ± 2.97	P10: 5.34 ± 4.16	>0.05
		P14: 5.63 ± 3.55	P14: 3.57 ± 3.40	>0.05
		P21: 2.67 ± 0.60	P21: 3.56 ± 0.90	>0.05
		P90: 109.90 ± 5.35	P90: 99.50 ± 3.57	>0.05
	Renin receptor mRNA	P2: 8.31 ± 2.06	P2: 8.51 ± 9.68	>0.05
		P6: 14.57 ± 2.06	P6: 6.21 ± 2.05	<0.05
		P10: 7.53 ± 2.01	P10: 3.72 ± 0.88	<0.05
		P14: 5.09 ± 1.27	P14: 3.33 ± 0.34	>0.05
		P21: 1.32 ± 0.24	P21: 1.47 ± 0.24	>0.05
		P90: 1.86 ± 1.12	P90: 0.83 ± 0.29	>0.05
	Angiotensin type I receptor mRNA	P2: 3.26 ± 0.74	P2: 3.09 ± 0.50	>0.05
		P6: 3.50 ± 0.50	P6: 3.26 ± 0.57	>0.05
		P10: 10.34 ± 0.95	P10: 4.82 ± 0.25	<0.05
		P14: 7.87 ± 0.25	P14: 5.69 ± 0.94	<0.05
		P21: 0.58 ± 0.08	P21: 0.91 ± 0.24	>0.05
		P90: 0.58 ± 0.16	P90: 0.70 ± 0.25	>0.05
	Angiotensin type II receptor mRNA	P2: 0.97 ± 0.17	P2: 1.02 ± 0.14	>0.05
		P6: 1.80 ± 2.16	P6: 0.84 ± 0.17	<0.05
		P10: 1.88 ± 2.22	P10: 0.86 ± 0.14	<0.05
		P14: 1.42 ± 0.16	P14: 0.67 ± 0.04	<0.05
		P21: 0.06 ± 0.04	P21: 0.10 ± 0.05	>0.05
		P90: 0.05 ± 0.03	P90: 0.02 ± 0.01	>0.05
	ACE1 mRNA	P6: 1.45 ± 0.41	P6: 0.99 ± 0.32	>0.05
		P10: 2.41 ± 0.58	P10: 3.46 ± 0.67	>0.05
		P14: 1.28 ± 0.23	P14: 1.72 ± 0.20	>0.05
	ACE2 mRNA	P6: 0.49 ± 0.09	P6: 1.10 ± 0.28	<0.05
		P10: 1.69 ± 0.23	P10: 5.87 ± 7.15	<0.05
		P14: 0.99 ± 0.17	P14: 2.12 ± 0.44	<0.05
	MAS1 mRNA proto-oncogene	P6: 0.12 ± 0.05	P6: 0.17 ± 0.09	>0.05
		P10: 0.09 ± 0.03	P10: 0.07 ± 0.05	>0.05
		P14: 0.12 ± 0.03	P14: 0.12 ± 0.05	>0.05
	Neprilysin mRNA	P6: 0.64 ± 0.29	P6: 0.99 ± 0.29	>0.05
		P10: 1.69 ± 0.46	P10: 1.89 ± 0.70	>0.05
		P14: 0.93 ± 0.20	P14: 0.73 ± 0.23	>0.05
	Aminopeptidase A	P6: 412.63 ± 29.41	P6: 357.27 ± 17.30	>0.05
		P10: 467.13 ± 34.60	P10: 355.54 ± 27.68	<0.05
		P14: 410.03 ± 15.58	P14: 359.86 ± 15.57	<0.05
	Angiotensin II (ng/mL/mg)	P2: 2.25 ± 0.24	P2: 2.95 ± 0.38	>0.05
		P6: 3.90 ± 0.72	P6: 8.65 ± 2.06	<0.05
		P10: 2.18 ± 0.22	P10: 3.19 ± 0.31	<0.05
		P14: 1.99 ± 0.41	P14: 3.33 ± 0.69	<0.05
		P21: 1.49 ± 0.50	P21: 2.28 ± 0.35	>0.05
		P90: 1.17 ± 0.17	P90: 0.93 ± 0.20	>0.05
	Plasma renin (ng/AngI/mL)	P2: 11.79 ± 2.68	P2: 8.75 ± 1.41	>0.05
		P6: 36.77 ± 6.07	P6: 12.72 ± 3.39	<0.05
		P10: 21.25 ± 2.68	P10: 12.61 ± 3.15	<0.05
		P14: 14.82 ± 3.16	P14: 7.35 ± 0.59	>0.05
		P21: 7.59 ± 0.70	P21: 6.89 ± 0.93	>0.05
		P90: 13.66 ± 3.03	P90: 11.67 ± 2.34	>0.05
Mahanes et al., 2020 [33]	Urinary angiotensinogen (ng/mL)	85.88 ± 4.18	72.17 ± 2.68	<0.05
	Renal cortex angiotensinogen mRNA (2^−ΔΔCt^)	0.90 ± 0.21	0.82 ± 0.12	>0.05
	Renal cortex angiotensinogen protein (ng/mL/mg)	112.24 ± 10.62	93.88 ± 12.24	>0.05

MS: Maternal separation; P: postnatal day; SE: Standard error; AR: adrenergic receptors.

**Table 8:** Some alterations induced by maternal separation in rodents and associated clinical findings in humans.

Type of Alteration	Findings in Rodents	Associated Clinical Manifestation in Humans
Morphological	Increase in MPO^+^, CD44^+^ cells in the glomeruli and TLR4^+^ cells in kidney [14]	IgA Nephropathy [55], focal segmental glomerulosclerosis [56] and, crescentic glomerulonephritis [57]
	Increased microvascular density [23]	Polycystic kidney disease [58]
Functional	Increased ACE1 and ACE2 [23]	Hypertension, renal fibrosis and, diabetic nephropathy [59]
	Reduction in antioxidant enzymes [16]	Increased oxidative stress, reduced glomerular filtration rates, and progression of chronic kidney disease [60]
Molecular	Increased renal IL-1β [14]	Renal inflammation associated with hyperuricemia [61]
	Increased mRNA of RAS receptors [23]	Renal inflammation and glomerulonephritis [62]
	Increased plasma renin [23]	Chronic hypertension, and renal injury [63]

## Data Availability

The data presented in this study are available on request from the corresponding authors.

## Abbreviations

The following abbreviations are used in this manuscript:

MS	Maternal separation
RAS	Renin-angiotensin system
P	Postnatal day
ROS	Reactive oxygen species
AR	Adrenergic receptors
